# Case Report: Differential outcomes associated with the same pathogenic variant: long-term follow-up of a CHARGE syndrome case with a nonsense mutation c.6292C>T in the CHD gene

**DOI:** 10.3389/fgene.2026.1932117

**Published:** 2026-08-31

**Authors:** Qiong-yu Wang, Jing-jing Huang, Fu-chun Wang, Jing-wei He

**Affiliations:** 1 Xiamen Children’s Hospital/Children’s Hospital of Fudan University at Xiamen, Xiamen, Fujian, China; 2 Children’s Hospital of Fudan University, Shanghai, China

**Keywords:** CHARGE syndrome, CHD7 gene, c.6292C>T nonsense pathogenic variant, early intervention, multidisciplinary management

## Abstract

**Background:**

CHARGE syndrome (OMIM #214800) is a rare autosomal dominant multisystem disorder, most commonly attributable to *de novo* heterozygous loss-of-function pathogenic variants in the *CHD7* gene. Pathogenic Pathogenic variants of *CHD7* are distributed throughout the entire coding region, with nonsense and frameshift pathogenic variants predominating, and the vast majority constitute private mutations. Owing to its broad phenotypic spectrum and marked variability in expressivity, early diagnosis remains challenging. This article presents the long-term follow-up data of a female patient with genetically confirmed CHARGE syndrome, with particular emphasis on her clinical trajectory and outcomes achieved through multidisciplinary management.

**Case presentation:**

The patient was a full-term female born via spontaneous vaginal delivery in 2017, presenting with respiratory distress and feeding difficulties immediately after birth. Comprehensive evaluation revealed multisystem abnormalities involving the respiratory, cardiovascular, neurological, and sensory systems. Trio-based whole-exome sequencing identified a *de novo* heterozygous nonsense pathogenic variant, c.6292(EXON31)C>T (p.Arg2098*), in the *CHD7* gene. Literature review indicated that this pathogenic variant had been previously reported in cases that all resulted in infantile death. In contrast, our patient, following active multidisciplinary management, achieved significant improvement in multiple organ functions and survived to school age, where she now adapts well to a special education school environment. This case provides the first evidence that this pathogenic variant is compatible with a favorable long-term outcome, highlighting substantial phenotypic heterogeneity and prognostic diversity even among individuals carrying an identical genetic alteration.

**Conclusion:**

This case highlights the critical importance of early genetic diagnosis and coordinated multidisciplinary management in CHARGE syndrome. By presenting divergent outcomes associated with the same pathogenic variant, our findings enrich the clinical evidence on *CHD7* genotype–phenotype correlations. Even with severe neonatal multisystem involvement, proactive and individualized management can achieve favorable long-term outcomes.

## 1 Introduction

CHARGE syndrome (OMIM #214800) is a rare congenital multisystem developmental malformation syndrome, with a neonatal incidence of approximately 1 in 10,000 to 1 in 15,000 live births (Blake and Hudson, 2017). The majority of cases are caused by *de novo* heterozygous pathogenic variants in the *CHD7* gene, following an autosomal dominant inheritance pattern with high penetrance and remarkable phenotypic heterogeneity. The syndrome was first reported by Hall in 1979 (Hall, 1979), and Pagon et al. (Pagon et al., 1981) first proposed the acronym in 1981, derived from the initial letters of its classical clinical features: Coloboma, Heart defects, Atresia of the choanae, Retarded growth and development, Genital hypoplasia, and Ear anomalies. Due to the complex and diverse clinical manifestations, the lack of specific early symptoms, and the fact that most affected infants often present with a single initial symptom such as respiratory distress, feeding difficulties, or hearing impairment, the rates of misdiagnosis and underdiagnosis remain exceedingly high in clinical practice (Hale et al., 2016; Zhang and Gong, 2019; Hsu et al., 2014). Furthermore, CHARGE syndrome may involve multiple systems, including the respiratory, cardiovascular, neurological, ophthalmological, and auditory systems, necessitating long-term coordinated multidisciplinary interventions involving neonatology, cardiology, otorhinolaryngology, rehabilitation medicine, ophthalmology, and other specialties. Management by a single specialty alone is insufficient to achieve standardized and comprehensive care throughout the entire clinical course (Dijk et al., 2019).

We report a case of CHARGE syndrome in a child harboring a *de novo* heterozygous nonsense pathogenic variant, c.6292(EXON31)C>T (p.Arg2098*), in the *CHD7* gene, as confirmed by whole-exome sequencing, with 7 years of longitudinal follow-up. To date, this specific pathogenic variant has been reported in three prior cases: a female infant reported by Gennery et al. (Gennery et al., 2008) who died of cardiopulmonary failure, a Chinese male neonate reported by Sun et al. (Sun et al., 2020) who died at 5 months of age, and an unnamed infant in a neonatal cohort study who could not be weaned from respiratory support and subsequently expired (Wang et al., 2023). All three patients died during infancy. Our patient, however, survived to school age with good functional outcomes after comprehensive multidisciplinary care, underscoring the considerable phenotypic heterogeneity and prognostic variability that may occur even with an identical genetic alteration. Drawing on complete longitudinal data, we systematically describe the patient’s clinical trajectory from neonatal presentation, multisystem anomalies, and staged surgical corrections to long-term rehabilitation. In conjunction with a review of the pertinent literature, we explore the clinical phenotype, pathogenic mechanisms, early diagnosis, and prognosis of CHARGE syndrome. Through the lens of disparate outcomes associated with the same pathogenic variant, this report enriches the current understanding of *CHD7* genotype–phenotype relationships and provides practical insights into early screening, precise molecular diagnosis, and individualized management of rare genetic disorders.

## 2 Case presentation

### 2.1 General information and perinatal history

The patient, a female infant, was delivered at term via spontaneous vaginal delivery on December 23, 2017. The maternal and paternal ages at delivery were 29 and 32 years, respectively. This was the mother^,^s first pregnancy (gravida1, para1). The parents were non-consanguineous, with no documented family history of inherited disorders, nor any history of maternal medication use, infection, or environmental exposure during gestation. Birth measurements included a length of 51 cm and a weight of 3.6 kg, with Apgar scores of 9 at 1, 5, and 10 min after birth.

### 2.2 Neonatal manifestations (from 1 hour to day 8 of life)

The patient was admitted to the Department of Neonatology at Xiamen Maternal and Child Health Hospital 58 min after birth due to tachypnea. Physical examination revealed that the patient was alert with fair responsiveness. She exhibited tachypnea, and auscultation of the lungs disclosed coarse breath sounds with audible rhonchi bilaterally. Cardiac examination demonstrated a regular rhythm, strong heart sounds, and an audible murmur. Oral examination showed a relatively small oral cavity with mild macroglossia. Pulmonary CT revealed possible membranous atresia of the left posterior choana, neonatal pneumonia, and stenosis of the lower trachea. Echocardiography demonstrated patent ductus arteriosus (0.23 cm), patent foramen ovale, and trace tricuspid regurgitation. Cranial MRI (performed on day 6 of life) showed bilateral subependymal hemorrhage, subarachnoid hemorrhage, and bilateral ventricular enlargement. Fundoscopic examination revealed yellowish-white lesions in the inferior retina bilaterally with vessels traversing the surface, and the optic discs were not visible, leading to a diagnosis of congenital chorioretinal coloboma. The discharge diagnoses included neonatal pneumonia, airway malformation, intracranial hemorrhage, congenital chorioretinal coloboma, and suspected choanal atresia.

### 2.3 Genetic testing and confirmation

**FIGURE 1:**
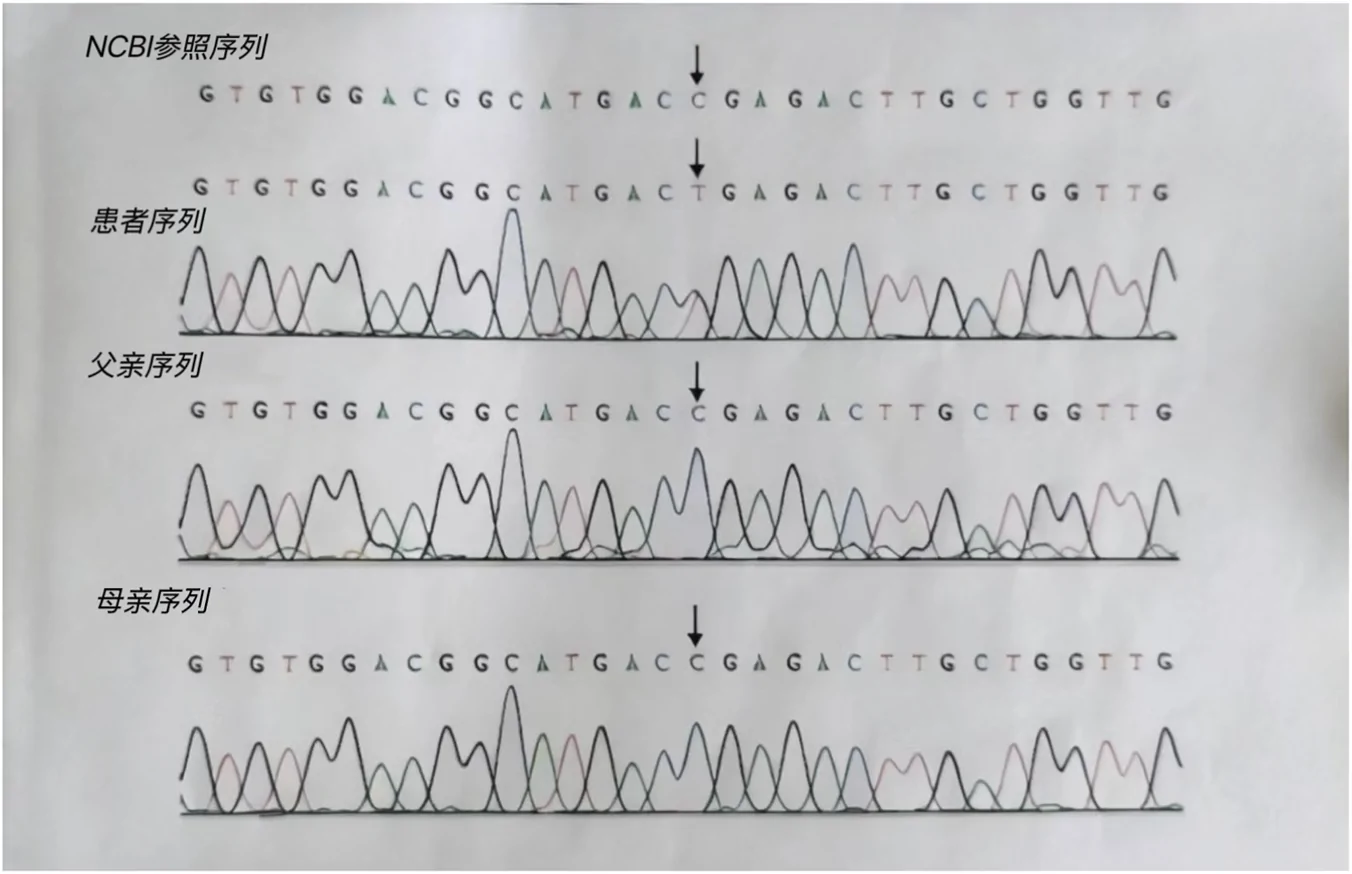
DNA sequencing chromatogram comparison showing four horizontal panels labeled NCBI reference sequence, patient sequence, father sequence, and mother sequence, with arrows marking a variant site in each panel for genetic analysis.

Following admission, the infant exhibited feeding difficulties and glossoptosis, accompanied by a small oral cavity and weak cry on examination. In view of the multisystem anomalies detected, a possible underlying genetic or metabolic disorder was suspected. Thus, trio-based whole-exome sequencing (WES) was performed on the 5th day after birth (December 28, 2017). In 2017, while WES was not yet a universal first-tier test for all neonatal patients, it was increasingly employed for complex cases with multisystem involvement suggestive of a genetic etiology, as it offered a higher diagnostic yield than targeted gene panels. The results, which became available 2 months later, identified a heterozygous nonsense pathogenic variant, c.6292(EXON31)C>T (p.Arg2098*), in the *CHD7* gene (Figure 1). This pathogenic variant was absent in both parents, confirming its *de novo* origin. Combined with the characteristic clinical features, a definitive diagnosis of CHARGE syndrome was made.

Following the genetic diagnosis, the family received comprehensive genetic counseling. At that time, they were counseled about the broad and often severe phenotypic spectrum associated with CHARGE syndrome, including potential life-threatening complications, feeding difficulties, hearing loss, and developmental delays, with a guarded prognosis based on the published outcomes of similar cases. The family experienced significant initial distress and anxiety. However, they chose to pursue aggressive supportive care. Over the subsequent 7 years, as the child achieved successive developmental milestones with multidisciplinary support, the family’s perspective gradually shifted from one of survival-focused worry to active participation in her rehabilitation and education, demonstrating remarkable resilience and adaptation.

### 2.4 Early intervention and multisystem assessment

Respiratory and Upper Airway: On day 8 of life, the infant was transferred to Guangdong Provincial People’s Hospital for further evaluation. Flexible bronchoscopy revealed glossoptosis, impaired epiglottic elevation, and narrowing of the right main bronchus, prompting revision of the diagnosis to congenital tracheal anomaly and congenital bronchial stenosis. In-hospital management included alternating lateral decubitus positioning and supplemental low-flow oxygen. Although the infant’s breath sounds improved, tachypnea persisted. After the parents received appropriate home-care training, the infant was discharged against medical advice. Post-discharge, feeding difficulties persisted due to weak sucking, necessitating nasogastric tube feeding for 6 months, after which the infant gradually transitioned to oral feeding. Nevertheless, the volume per feed remained low, and weight gain was suboptimal.

Cardiovascula: The patient was diagnosed with secundum atrial septal defect, severe pulmonary arterial hypertension, and patent ductus arteriosus. At the age of 3 years and 5 months (June 2, 2021), she successfully underwent percutaneous patent ductus arteriosus closure. Postoperatively, cardiac function returned to normal, with only a patent foramen ovale remaining.

Growth and Neurodevelopment: Motor milestones were significantly delayed: the patient could sit with instability at 1 year of age, did not walk independently until 2 years of age with unsteady gait, and was unable to jump off the ground at 3 years and 1 month of age. Regular rehabilitation training, including motor therapy and speech training, was implemented. Language development delay improved markedly following cochlear implantation. The patient’s height growth curve was at the borderline of the 3rd percentile, showing a fluctuating growth pattern. At 8 years and 3 months of age (April 3, 2026), she was referred to our department due to slow height gain. Her height was measured at 118 cm (< 3rd percentile). After a 2-month comprehensive intervention comprising traditional Chinese medicine non-pharmacological therapies, along with dietary, sleep, and exercise management, her height increased by approximately 0.5 cm.

Ophthalmology and Vision: The bilateral chorioretinal colobomas persisted throughout follow-up; however, refractive development was within normal limits, and the patient did not exhibit significant visual impairment.

Otology and Hearing: Temporal bone CT performed at 7 months of age revealed bilateral Mondini malformations, consistent with inner ear dysplasia, accompanied by mastoiditis. Audiological screening at 11 months was unsuccessful. Bilateral hearing aids were prescribed at 1 year and 2 months of age, resulting in some improvement in auditory responsiveness, albeit still below age-appropriate norms. At 19 months, MRI confirmed bilateral semicircular canal agenesis and bilateral hypoplasia of the auditory nerves. Left cochlear implantation was performed at 21 months of age (September 26, 2019), followed by gradual improvement in hearing function. By the age of 6 years and 11 months, with the combined use of a right hearing aid and the left cochlear implant, the patient passed the hearing screening test.

### 2.5 Recent follow-up

At 6 years and 11 months of age (December 2024), cardiac ultrasound revealed that the occluder was well positioned without evidence of residual shunt. Although mild developmental delay remained, it had notably improved relative to the infant and toddler period. Hearing function was adequately compensated, allowing effective daily communication. The child is currently attending a hearing-impaired class at a special education school, achieving a moderate level of academic performance.

## 3 Discussion

CHARGE syndrome is a multisystem congenital disorder characterized by marked phenotypic heterogeneity. Respiratory distress and feeding difficulties are frequently the initial manifestations in the neonatal period (Pagon et al., 1981). The present case fulfilled three major diagnostic criteria—chorioretinal coloboma, congenital airway anomalies, and inner ear malformations—along with several minor features, including cardiac defects, growth retardation, and feeding difficulties. The diagnosis was confirmed by the identification of a pathogenic variant in *CHD7*. Nevertheless, given that CHARGE syndrome seldom presents with the complete spectrum of classic features and often shows atypical combinations, affected neonates are prone to misdiagnosis as having isolated congenital heart disease, pneumonia, or hearing disorders (Hale et al., 2016). Thus, for neonates presenting with unexplained multiorgan anomalies, early genetic evaluation is warranted to prevent delays in the diagnosis and appropriate management of the underlying disorder.

Our patient is a child with CHARGE syndrome who harbors a *de novo* heterozygous nonsense pathogenic variant, c.6292(EXON31)C>T (p.Arg2098*), in the *CHD7* gene. This pathogenic variant creates a premature stop codon at position 2098, leading to a truncated protein product and a loss-of-function allele (Sun et al., 2020). The *CHD7* gene, located on chromosome 8, encodes chromodomain helicase DNA-binding protein 7, which belongs to the ATP-dependent chromatin remodeling family. By modulating chromatin accessibility and transcriptional activity of multiple tissue-specific genes during embryogenesis, *CHD7* is essential for the proper development of the respiratory, cardiovascular, visual, auditory, nervous, and craniofacial systems (Vissers et al., 2004; Woodag et al., 1997). Heterozygous loss-of-function pathogenic variants of *CHD7* cause dysregulation of downstream target genes through haploinsufficiency, resulting in impaired multisystem differentiation during embryonic development. This mechanistic framework underpins the substantial phenotypic heterogeneity and multisystem manifestations observed in CHARGE syndrome. Pathogenic variants in *CHD7* are scattered across the entire coding sequence, with nonsense and frameshift pathogenic variants accounting for the majority (approximately 70%), and most are private mutations (Lalani et al., 2006).

A review of the literature indicated that this pathogenic variant is not without prior documentation; it has been cataloged in international genetic databases (ClinVar pathogenic accession: VCV000226115; dbSNP: rs875989879), confirming its established pathogenicity in CHARGE syndrome. Specifically, three previously reported patients harboring this identical pathogenic variant all died during infancy: (1) a female case reported by Gennery et al. (Gennery et al., 2008) who had persistent truncus arteriosus and immunodeficiency and died of cardiopulmonary failure; (2) a Chinese male neonate reported by Sun et al. (Sun et al., 2020) presenting with atrial septal defect, optic disc aplasia, semicircular canal hypoplasia, and cerebral hypoplasia, who died of infection and malnutrition at 5 months of age; and (3) an infant in a neonatal cohort study who could not be weaned from respiratory support and subsequently expired (Wang et al., 2023). By contrast, our patient, having received systematic multidisciplinary care, survived to school age with a good functional outcome, representing a markedly different clinical course from previously reported cases with the same genetic alteration. This case provides the first evidence that favorable long-term outcomes can be achieved with this pathogenic variant, underscoring the considerable phenotypic heterogeneity and prognostic diversity that may be observed even among individuals carrying an identical pathogenic variants, and contributing valuable data to the expanding body of evidence on *CHD7* genotype–phenotype relationships.

**FIGURE 2:**
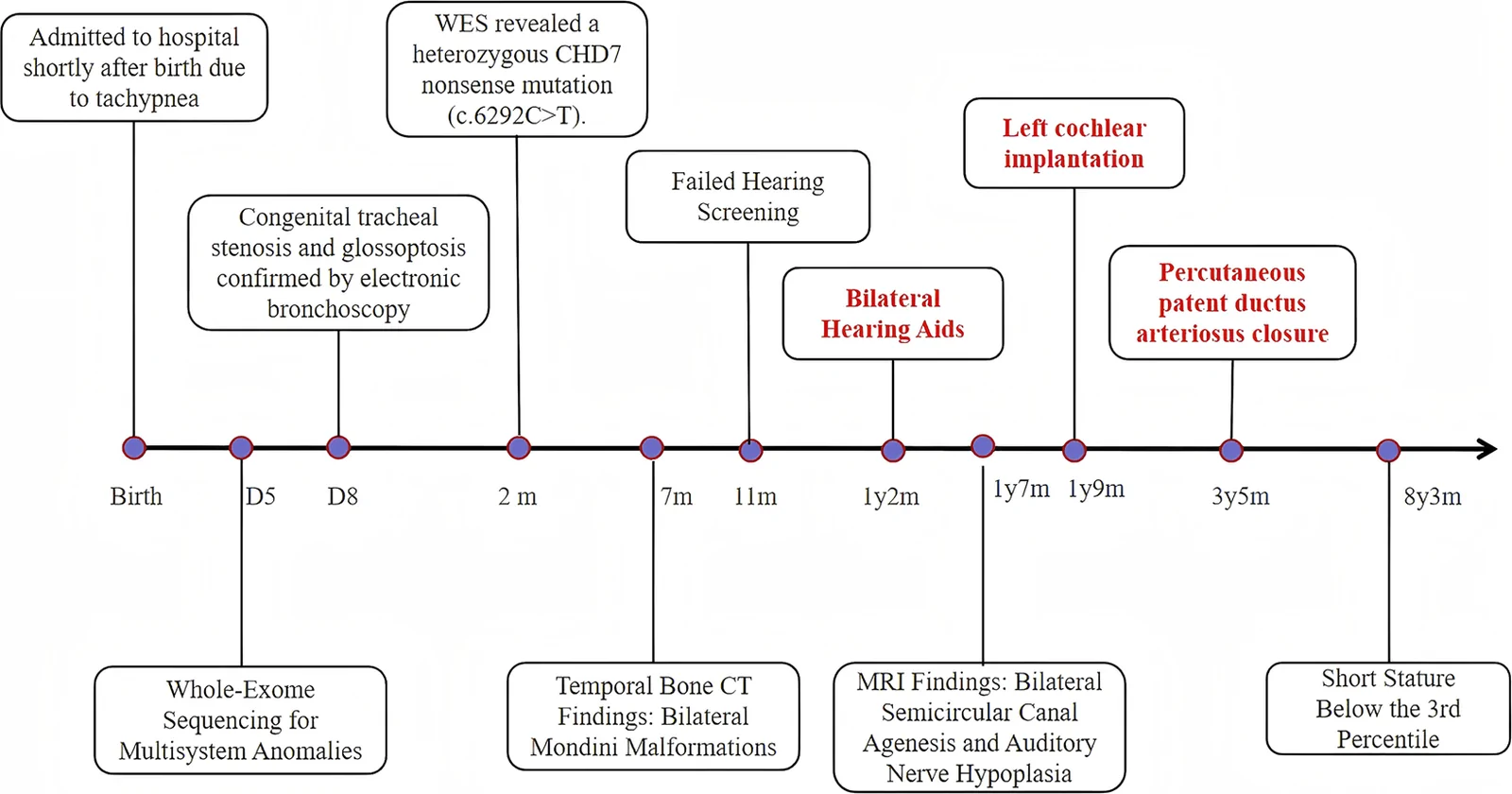
Horizontal medical timeline diagram showing key clinical events from birth to 8 years 3 months, including neonatal hospitalization for tachypnea, diagnosis of tracheal stenosis, CHD7 mutation, failed hearing screening, hearing aids, left cochlear implantation, ductus arteriosus closure, imaging findings, and short stature.

What accounts for the striking prognostic heterogeneity associated with an identical genetic pathogenic variant across different individuals? The longitudinal follow-up of our patient over 7 years offers valuable insights. During the neonatal period, our patient demonstrated multisystem involvement encompassing the airway (congenital tracheal stenosis and glossoptosis), the cardiovascular system (atrial septal defect, patent ductus arteriosus with severe pulmonary arterial hypertension), the eyes (bilateral chorioretinal coloboma), the inner ear (Mondini malformations, bilateral semicircular canal aplasia, and hypoplastic auditory nerves), and the nervous system (intracranial hemorrhage and ventriculomegaly). The breadth of system involvement was not substantially different from that documented in previously reported cases. Nevertheless, the distinguishing feature of our case resides in the prompt implementation of a systematic multidisciplinary assessment and intervention strategy—trio-based whole-exome sequencing was requested on day 5 of life; transfer to a higher-level facility for flexible bronchoscopy was accomplished on day 8; cochlear implantation was performed at 21 months; percutaneous closure of the patent ductus arteriosus was completed at 3 years and 5 months; and the patient has undergone uninterrupted hearing, speech, and motor rehabilitation extending into the school-age years. To clearly illustrate this comprehensive clinical course and the timing of key interventions, we have summarized the patient’s diagnostic and therapeutic milestones in a timeline figure (Figure 2). The sustained multidisciplinary approach implemented in this case suggests that, in addition to the genetic predisposition conferred by the underlying pathogenic variant, early definitive diagnosis and systematic multidisciplinary care serve as critical modifiable factors that shape long-term prognosis. The previously reported cases, all of which lacked comprehensive medical support of comparable intensity and continuity to that provided in the present case, may partially account for the prognostic discrepancies observed.

Beyond its multisystem manifestations, growth retardation is a cardinal feature of CHARGE syndrome. It has been reported that approximately 10% of affected individuals have concurrent growth hormone deficiency (GHD) (Costa et al., 2020). Dörr et al. (Dörr et al., 2015) analyzed 51 patients with CHARGE syndrome treated with recombinant human growth hormone (rhGH) using data from the Pfizer International Growth Database (KIGS) and showed that after a median treatment period of 2.7 years, height standard deviation score (SDS) improved from −3.6 pretreatment to −2.2. In a Polish cohort of 29 patients with molecularly confirmed *CHD7* pathogenic variants, only two patients with GHD received rhGH; despite an improvement in growth velocity following treatment, their heights failed to reach the 3rd percentile (Lubońska-Cebula and Śmigiel, 2025). These studies consistently suggest that, despite rhGH therapy, most patients remain below the normal growth chart, and although treatment may enhance growth rate to a certain degree, complete catch-up growth is seldom attainable. In the present case, the response to non-pharmacological traditional Chinese medicine interventions combined with lifestyle modifications was unsatisfactory. Hence, regular anthropometric monitoring is essential, and growth hormone stimulation testing should be considered when clinically warranted to rule out GHD. Should GHD be confirmed, rhGH therapy could be initiated, with meticulous follow-up of both growth velocity and height catch-up.

At present, no curative therapy is available for CHARGE syndrome. Early and accurate genetic diagnosis, multidisciplinary and individualized intervention, together with long-term rehabilitative follow-up, constitute the cornerstone of prognosis improvement (Köhler et al., 2025). The favorable long-term outcome of our patient, who received systematic multidisciplinary care spanning from the neonatal period to school age, provides real-world clinical evidence in support of this integrated approach. Respiratory distress and feeding difficulties are among the earliest challenges encountered in neonates with CHARGE syndrome (Gao et al., 2025). In the present case, glossoptosis, impaired epiglottic elevation, and tracheal stenosis resulted in severe sucking dysfunction, requiring nasogastric tube feeding for 6 months. This experience highlights the importance of early flexible bronchoscopy for airway evaluation and the formulation of individualized nutritional regimens in patients with choanal atresia or tracheal anomalies, so as to prevent aspiration pneumonia and malnutrition, and to establish a solid foundation for subsequent therapeutic interventions. From a cardiovascular perspective, our patient presented with patent ductus arteriosus and severe pulmonary arterial hypertension, which rendered direct transcatheter closure a high-risk procedure. Following a period of dynamic clinical surveillance, interventional occlusion was successfully performed at 3 years and 5 months of age, effectively mitigating the risk factors for pulmonary hypertension. This case illustrates that a staged, assessment-guided treatment approach can significantly reduce perioperative risk. With respect to auditory rehabilitation, inner ear malformations—including Mondini malformations and semicircular canal aplasia—are frequently observed in CHARGE syndrome. In our patient, MRI at 19 months confirmed bilateral semicircular canal agenesis and hypoplastic auditory nerves. After suboptimal benefit from hearing aids, left cochlear implantation was performed at 21 months of age, followed by marked improvement in auditory function. This supports the notion that early cochlear implantation (at 1–2 years of age) constitutes an effective intervention for hearing restoration in patients with inner ear structural anomalies and auditory nerve hypoplasia (Wang et al., 2022; Kay-Rivest et al., 2022). In terms of neurodevelopment, despite a history of neonatal intracranial hemorrhage and ventriculomegaly, the patient attained cognitive abilities at the average level for her age at 6 years and 11 months. Although motor development lagged behind age norms, steady improvement was achieved through ongoing rehabilitation. These observations indicate that the neurodevelopmental trajectory of CHARGE syndrome exhibits substantial individual variation, and that early institution of rehabilitation, coupled with sustained long-term training, plays an indispensable role in enhancing motor, linguistic, and social competencies. Future studies should incorporate standardized neurocognitive assessments to generate objective quantitative data, thereby strengthening the evidence base for life-span management of such complex multisystem chronic conditions.

Regarding other *CHD7* variants, while direct genotype-phenotype correlations remain challenging due to the high prevalence of private mutations, some reports have described long-term survivors with severe multisystem involvement. For instance, patients with certain missense variants or those with variants in regions outside the helicase domains have occasionally been reported with milder phenotypes or better survival. Costa et al. (Costa et al., 2020) reported a female patient with a *CHD7* frameshift variant (c.2509_2512delCATT) who survived to 10.3 years of age with early growth hormone therapy. However, to our knowledge, a comparable long-term follow-up to school age with favorable functional outcomes, specifically for a truncating variant like p.Arg2098*, has not been previously documented. Our case thus provides a unique and valuable addition to the literature.

## 4 Conclusion

We present a case of classic CHARGE syndrome in a child attributable to a *de novo* nonsense pathogenic variant, c.6292(EXON31)C>T (p.Arg2098*), in the *CHD7* gene, whose clinical manifestations involved multiple systems, including the cardiovascular, respiratory, ophthalmologic, auditory, and neurological systems. Following a comprehensive management strategy comprising respiratory support and early genetic confirmation in the neonatal period, auditory rehabilitation with cochlear implantation at 21 months of age, percutaneous cardiac intervention at 3 years and 5 months, and sustained multidisciplinary follow-up over a period of 7 years, the patient achieved survival to school age with favorable functional outcomes.

Notably, among the three previously documented patients carrying this identical pathogenic variant—a female with persistent truncus arteriosus and immunodeficiency who died of cardiopulmonary failure, a Chinese male neonate who died of infection and malnutrition at 5 months of age, and an infant in a cohort study who could not be weaned from respiratory support—all succumbed during infancy. In stark contrast, our case provides the first demonstration that a favorable long-term prognosis is achievable with this pathogenic variant. This observation highlights that marked phenotypic heterogeneity and prognostic diversity may be observed even among individuals with the same genetic alteration, and that early accurate diagnosis coupled with systematic multidisciplinary management constitutes the key to optimizing outcomes. Furthermore, our patient had significant growth retardation that responded poorly to traditional Chinese medicine non-pharmacological interventions and lifestyle modifications. Accordingly, regular anthropometric monitoring is recommended, and growth hormone stimulation testing should be considered when clinically warranted to rule out GHD. Should a diagnosis of GHD be confirmed, rhGH therapy could be initiated, with meticulous follow-up of growth velocity and height catch-up.

CHARGE syndrome is notable for its complex clinical heterogeneity. Whole-exome sequencing represents the cornerstone of accurate genetic diagnosis. Multidisciplinary collaborative care, timely surgical intervention, and sustained individualized rehabilitation with long-term follow-up constitute the essential elements for optimizing growth, developmental outcomes, and long-term quality of life in affected individuals. By focusing on divergent prognostic trajectories associated with an identical pathogenic variant, this case expands the existing body of evidence regarding *CHD7* genotype–phenotype correlations and offers practical insights into the early recognition and comprehensive management of rare genetic disorders.

## Data Availability

The datasets presented in this article are not readily available because of ethical and privacy restrictions. Requests to access the datasets should be directed to the corresponding authors.
