# Supplementary material for: Case Report: Differential outcomes associated with the same pathogenic variant: long-term follow-up of a CHARGE syndrome case with a nonsense mutation c.6292C>T in the CHD gene
Source: Front Genet. 2026 Aug 31;17:1932117. doi: 10.3389/fgene.2026.1932117 (PMC13571904; doi:10.3389/fgene.2026.1932117)
Supplement: Supplementary file 1 [file Supplementaryfile1.doc]

## CARE Checklist

**Title:**

The title identifies the manuscript as a case report of CHARGE syndrome, specifies the genetic variant (CHD7 gene c.6292C>T nonsense mutation), and highlights the unique longitudinal follow-up and differential prognostic outcomes.

**Keywords:**

Key diagnoses and features are listed in the Keywords section, including CHARGE syndrome, CHD7 gene, c.6292C>T nonsense variant, early intervention, and multidisciplinary management.

**Abstract:**

The Abstract summarises what is unique about this case, namely the seven-year longitudinal follow-up of a female patient with CHARGE syndrome carrying a CHD7 c.6292C>T nonsense variant, who survived to school age with favourable functional outcomes despite all previously reported cases with the same variant having succumbed in infancy. It outlines the patient's multisystem involvement, diagnostic confirmation via trio-based whole-exome sequencing, multidisciplinary interventions, and the key clinical lessons regarding phenotypic heterogeneity and prognostic variability.

**Introduction:**

The Introduction explains the background to CHARGE syndrome, including its aetiology (CHD7 loss-of-function variants), clinical heterogeneity, diagnostic challenges, and the need for multidisciplinary management. It clarifies why this longitudinal case with an identical variant but divergent outcome provides novel and clinically relevant insight into genotype-phenotype correlations.

**Patient Information:**

De-identified patient information, including the genetic finding (CHD7 c.6292C>T nonsense variant, de novo), perinatal history, and relevant medical background, is presented in the Case Presentation section. Family history (non-consanguineous parents, no family history of inherited disorders) and psychosocial context are described where relevant.

**Clinical Findings:**

Significant physical, neurological, ophthalmological, audiological, cardiovascular, and respiratory findings are described in the Clinical Findings section, including tachypnoea, congenital chorioretinal coloboma, choanal atresia, congenital tracheal and bronchial anomalies, patent ductus arteriosus, atrial septal defect, pulmonary arterial hypertension, bilateral Mondini malformations, semicircular canal agenesis, hypoplastic auditory nerves, neonatal intracranial haemorrhage, ventriculomegaly, and significant motor and language developmental delay.

**Timeline:**

Historical and current clinical information is organised in a chronological narrative within the Case Presentation section, summarising key diagnostic, surgical, and developmental milestones from birth (December 2017) through to school age (April 2026), including genetic testing on day 5 of life, cochlear implantation at 21 months, percutaneous cardiac intervention at 3 years and 5 months, and ongoing rehabilitation.

**Diagnostic Assessment:**

Diagnostic methods, including trio-based whole-exome sequencing, flexible bronchoscopy, pulmonary CT, temporal bone CT, cranial MRI, echocardiography, fundoscopy, and audiological assessments, are described in the Diagnostic Assessment section. Diagnostic challenges, including the initial suspicion of isolated congenital anomalies and the eventual genetic confirmation, are discussed.

**Therapeutic Interventions:**

Therapeutic and supportive interventions, including respiratory support (positioning, low-flow oxygen), nasogastric tube feeding for six months, patent ductus arteriosus percutaneous closure, left cochlear implantation, motor and speech rehabilitation, traditional Chinese medicine non-pharmacological therapies, and dietary/sleep/exercise management, are described in the Therapeutic Interventions section.

**Follow-up and Outcomes:**

Longitudinal follow-up and outcomes are described in the Follow-up and Outcomes section, including functional changes associated with hearing compensation, cardiac recovery, neurodevelopmental progress, growth trajectory, and the patient's current attendance at a special education school with moderate academic performance.

**Discussion:**

The Discussion provides a scientific analysis of the case in the context of existing literature, addressing genotype-phenotype correlations, the striking prognostic heterogeneity associated with the same variant, the mechanistic role of CHD7 haploinsufficiency, and the importance of early genetic diagnosis and multidisciplinary management. Strengths and limitations of the case report are considered, and the rationale for the conclusions is explained.

**Patient Perspective:**

Not included. Owing to the patient's young age and developmental status, it was not possible to obtain a meaningful direct patient perspective. Accordingly, the case is presented through clinical records and caregiver observations.

**Informed Consent:**

Written informed consent for publication of clinical information and images was obtained from the patient's legal guardian, as stated in the Ethics statement and Ethics approval and consent to participate sections. The study was approved by the Ethics Committee of Xiamen Children's Hospital (No. XCSER [2022] 42).
